# Macrophage Bactericidal Activities against *Staphylococcus aureus* Are Enhanced *In Vivo* by Selenium Supplementation in a Dose-Dependent Manner

**DOI:** 10.1371/journal.pone.0135515

**Published:** 2015-09-04

**Authors:** Mourad Aribi, Warda Meziane, Salim Habi, Yasser Boulatika, Hélène Marchandin, Jean-Luc Aymeric

**Affiliations:** 1 Laboratory of Applied Molecular Biology and Immunology, Department of Biology, University of Tlemcen, 13000, Tlemcen, Algeria; 2 Université Montpellier 1, UMR 5569 HydroSciences Montpellier, Équipe Pathogènes Hydriques Santé Environnements, 34093, Montpellier, Cedex 5, France; 3 Centre Hospitalier Régional Universitaire, Laboratoire de Bactériologie, 34295, Montpellier, Cedex 5, France; 4 UM2-INRA, UMR1333, Laboratoire Diversité, Génomes et Interactions Microorganismes Insectes, Université de Montpellier, Bataillon, 34095, Montpellier, Cedex 05, France; University of Colorado School of Medicine, UNITED STATES

## Abstract

**Background:**

Dietary selenium is of fundamental importance to maintain optimal immune function and enhance immunity during infection. To this end, we examined the effect of selenium on macrophage bactericidal activities against *Staphylococcus aureus*.

**Methods:**

Assays were performed in golden Syrian hamsters and peritoneal macrophages cultured with *S*. *aureus* and different concentrations of selenium.

**Results:**

Infected and selenium-supplemented animals have significantly decreased levels of serum nitric oxide (NO) production when compared with infected but non-selenium-supplemented animals at day 7 post-infection (*p* < 0.05). A low dose of 5 ng/mL selenium induced a significant decrease in macrophage NO production, but significant increase in hydrogen peroxide (H_2_O_2_) levels (respectively, *p* = 0.009, *p* < 0.001). The NO production and H_2_O_2_ levels were significantly increased with increasing concentrations of selenium; the optimal macrophage activity levels were reached at 20 ng/mL. The concentration of 5 ng/mL of selenium induced a significant decrease in the bacterial arginase activity but a significant increase in the macrophage arginase activity. The dose of 20 ng/mL selenium induced a significant decrease of bacterial growth (*p* < 0.0001) and a significant increase in macrophage phagocytic activity, NO production/arginase balance and *S*. *aureus* killing (for all comparisons, *p* < 0.001).

**Conclusions:**

Selenium acts in a dose-dependent manner on macrophage activation, phagocytosis and bacterial killing suggesting that inadequate doses may cause a loss of macrophage bactericidal activities and that selenium supplementation could enhance the *in vivo* control of immune response to *S*. *aureus*.

## Introduction

*S*. *aureus* is one of the most frequently isolated pathogens that often cause severe nosocomial infections, such as bloodstream infections, skin and soft tissue infections, and pneumonia. It is also responsible for a variety of suppurative infections and toxin-mediated diseases [1]. The nasal carriage has been associated with several infections including bacteremia, postoperative and diabetic foot ulcer infections, subjects colonized with *S*. *aureus* being at greater risk of subsequent infection than uncolonized individuals [2].

Macrophages are the primary professional scavenger cells. They can engulf microorganisms, proteins and other smaller cells using several mechanisms, such as Fc receptor- and complement-mediated phagocytosis, pinocytosis and endocytosis [3]. Macrophages are known to produce various molecules such as nitric oxide (NO) and reactive oxygen species (ROS) in response to phagocytosis and ligands of pattern recognition receptors (PRRs) [4]. The production of ROS and reactive nitrogen species (RNS) radicals are under the control of nicotinamide adenine dinucleotide phosphate oxidase (NOX) and inducible nitric oxide synthase (iNOS), respectively [5].

Activated macrophages produce NO by inducible NO synthase (iNOS), encoded by the NOS2 gene [6], by two successive monooxygenations of L-arginine (L-Arg) [7]. Hydrogen peroxide (H_2_O_2_) is one of the most active oxygen species, which is produced in the mitochondria by MnSOD (manganese-containing superoxide dismutase, SOD2) as an end product of plasma membrane associated-reduced nicotinamide adenine dinucleotide phosphate (NADPH) oxidase during the respiratory burst in activated macrophage [8]. Both NO and H_2_O_2_ play an important role as cell-signaling molecules and are effector agents for the microbicidal and cytotoxic response of macrophages after stimulation [9]. *S*. *aureus* cells can protect themselves against microbicidal agents generated by phagocytes by the expression of numerous virulence factors that allow them to colonize host tissues, to proliferate, and to escape the killing effect of the immune system [10].

Of note, *S*. *aureus* can survive intracellularly within phagocytes including neutrophils [11] and macrophages [12], and consequently evade host defenses and antibiotic treatment. Among *S*. *aureus* resistance mechanisms to phagocytosis, its ability to cleave the heavy chains of opsonic antibodies, using V8 protease (staphylococcal serine protease A, SspA) has been described [13]. This and other proteolytic enzymes may also be able to degrade the host antimicrobial peptide agents and tissue components [14]. The pathogenicity of *S*. *aureus* can also be linked to its ability to produce arginase. *S*. *aureus* arginase production modulates the immune system by consumption of the host arginine, resulting in reduced substrate for iNOS, thereby generating reduced amount of NO [15].

In the context of translational medicine, new therapeutic strategies against infectious pathogens using trace elements like selenium have been suggested to counteract pathogen immune evasion. Hence, selenium was used to prevent bacterial colonization on biomaterial surfaces [16] and *S*. *aureus* biofilm formation [17]. Supplementation with this micronutrient is usually required for optimum immune response through several enzymes, known as selenoproteins, involved in both innate and adaptive immunity [18]. Here, we tested the role of selenium supplementation on macrophage activities during *S*. *aureus* infection.

## Materials and Methods

### Ethics statement

The study was carried out with Good Laboratory Practices (GLP), and was reviewed and approved by the Institutional Ethics Committee of Tlemcen University. The necessary measures have been planned to prevent and minimize potential animal suffering if pain and distress are observed. In order to assess health and welfare, animals were regularly monitored and ongoing reviews of several criteria of such discomforts were used, including poor grooming, anorexia, decrease in water consumption, dehydration, decreased urination or stool output, vomiting or diarrhea, weight loss or loss of body condition, disorder in body temperature, disorder in pulse or respiratory rate, etc.

### *Staphylococcus aureus* strain

The *S*. *aureus* isolate was obtained from the Neonatology Department of Tlemcen University Medical Centre, Algeria. The isolate was a methicillin-susceptible *S*. *aureus* (MSSA) as determined by both phenotypic, *i*.*e*., disk diffusion method using oxacillin (5 mg) and cefoxitin (30 mg) disks (Bio-Rad) performed and interpreted according to recommendations of the Antibiogram Committee of the French Society of Microbiology, and genotypic methods performed as described previously [19] and showing no amplification of the *mecA* gene (S1 Fig).

### *In vivo* assays: animals, selenium supplementation and infection methods

A total of eighty (80) male golden Syrian hamsters (*Mesocricetus auratus*), six-week of age, weighing 60–80 g, were enrolled for a cross-sectional case/control study over six weeks. Hamsters were randomly divided into four equal groups; group 1, SA+/Se+ (n = 20, infected by the MSSA isolate and supplemented with selenium); group 2, SA+/Se- (n = 20, infected by the MSSA isolate and not supplemented with selenium); group 3, SA-/Se+ (n = 20, not infected but supplemented with selenium); group 4, SA-/Se- (n = 20, not infected and not supplemented with selenium, control group). Each group was divided into four subgroups of five hamsters to assess the concentration of circulating nitric oxide in regular time intervals and the levels of immunoglobulins and specific antibodies to evaluate the *in vivo* effect of selenium supplementation on feedback loop regulating immune response. Groups 1 and 2 were infected at the second week by intraperitoneal injection of *S*. *aureus* in a dose of 1 X 10^7^ CFU per mL in 1 mL sterile physiological saline with incomplete Freund's adjuvant. The control group was injected with 1 mL of 0.9% sterile physiological saline. Animals were monitored for clinical signs of *S*. *aureus* infection, such as hunched posture and reduced activity [20]. No mortality was recorded. In addition to clinical symptoms, infection was checked by microbiological culture procedure on Chapman medium [21]. The animals did not show signs of pain or distress during the experiment, except for the infection signs seen quite early. Intraperitoneal fluid samples were collected from the third day post-infection to isolate *S*. *aureus* and quantify bacterial load in infected groups (SA+/Se+ and SA+/Se-). Selenium supplementation was performed by oral administration of sodium selenite [22], at 0.6 mg/kg/day [23–25]. The amount of selenium in the basal diet was 0.1 mg/kg/day feed as minimal nutrient needs for animals [26]. Supplementation began 2 weeks before infection and continued until 1 day before sacrifice. On days 7, 14, 21 and 28 post-infection, the animals were weighed, and blood samples and intraperitoneal macrophages were collected. For the animal sacrifices, hamsters were euthanized with an overdose of intraperitoneal injection of sodium pentobarbital (150 mg/kg).

### Study design

**Fig 1:**
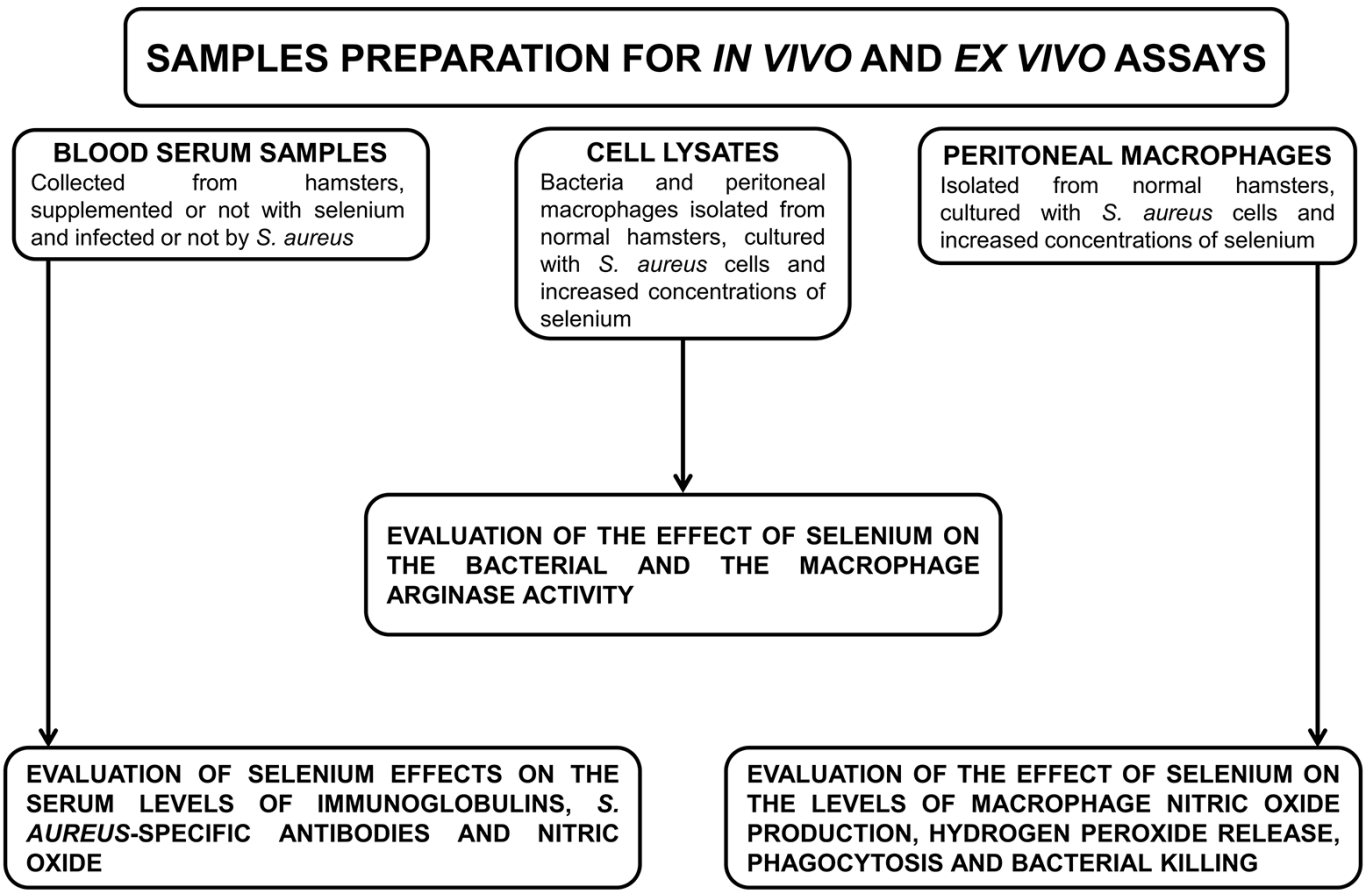
Study flow-chart.

Our experiments were carried out on serum samples, cell lysates and peritoneal macrophages (Fig 1). The evaluation of selenium effects on the serum levels of immunoglobulins, *S*. *aureus*-specific antibodies and NO was carried out in hamsters, supplemented (Se+) or not (Se-) with selenium and infected (SA+) or not (SA-) by *S*. *aureus*. Bacterial and macrophage lysates were used to measure the effect of selenium on the bacterial and the macrophage arginase activity, respectively. The effect of selenium on the levels of macrophage NO production, H_2_O_2_, bacterial growth, phagocytosis and bacterial killing were performed on a mixture of macrophages (2 × 10^6^ cells/mL) from normal hamsters, cultured with *S*. *aureus* cells (2 × 10^7^ CFU/mL) and diverse concentrations of selenium (0, 5, 10, 20, 30 and 40 ng/mL). The *ex vivo* macrophage activation was performed using the NO production and H_2_O_2_ assays. Fetal bovine serum (FBS) used in *ex-vivo* assays was quantified for each selenium level added to the medium. Each experiment was repeated at least four times.

### Sample preparation

For the preparation of peritoneal macrophages, hamster macrophages were aseptically harvested from the peritoneal cavity at various time-points by lavage with at least 10 mL sterile ice-cold phosphate-buffered saline (PBS) twice, injected intraperitoneally with gentle abdominal massage, as described elsewhere [27,28]. Macrophages were enriched after the incubation of total peritoneal cells harvested for 2 hours at 37°C to allow macrophages to adhere to tissue culture plastic [28–30]. Macrophages were seeded at 2 × 10^6^ cells/mL in cell culture media. Cells were counted using a hemocytometer under microscopy [31]. Macrophage cells were cultured as reported [32] in Dulbecco's modified Eagle's medium-high glucose (DMEM-HG, Sigma, Germany) with L-glutamine and supplemented with 10% (vol/vol) FBS, without antibiotics.

For the bacterial lysis assay, *S*. *aureus* was initially cultured using Brain-Heart Infusion Broth (BHIB) enrichment medium. Nine test tubes each containing 10 mL of BHIB medium were prepared, and 5, 10, 20, 30 and 40 ng/mL of selenium standard solution were respectively added to five of them. Then, each of the 9 tubes were inoculated with a bacterial suspension, and incubated at 37°C for 24 hours. In the next step, the bacterial cells were lysed. To this end, cells were firstly centrifuged at 20,000 x *g* for 20 min. After removal of the supernatants, the pellets were washed at least 3 times, to remove any remaining culture medium until the medium became colorless. The wash was performed by adding to the cell pellet, 1 mL of PBS buffer, and then continued by centrifugation at 1000 x *g* for 10 min. The pellet was suspended in 1 mL of lysis solution, 1% Triton X-100, 10 mM Tris-HCl pH 7. After a rigorous mixing followed by centrifugation at 20,000 x *g* for 20 min, the bacterial lysate was collected from the supernatant and protein content was measured. For the macrophage lysis assay, peritoneal macrophages were washed three times with 1.0 mL of PBS and then lysed by exposure to ice-cold 1% Triton X-100 buffer as reported [33,34]. The protein content of the supernatants was then measured.

### Experimental procedures

#### Protein assay

Protein concentration was measured at 540 nm by the colorimetric Biuret method using Thermo Scientific kit (Thermo Fisher Scientific Inc., Middletown, USA).

#### Immunoglobulins and specific antibodies assays

Total serum IgM and IgG antibody levels were measured quantitatively by agarose gel single radial immunodiffusion (SRID) assay using anti-golden Syrian Hamster IgG (goat, H + L) and IgM (rabbit, *μ*-chain specific) (Rockland Immunochemicals Inc., Gilbertsville, PA, USA), and respective isotype controls from serum of normal hamsters (BioLegend, San Diego, CA, USA). Specific antibodies were detected spectrophotometrically by ELISA reader at 490 nm using cell lysate, *S*. *aureus* antigen and plates treated with peroxidase-conjugated anti-IgG or anti-IgM as described [35].

#### Nitric oxide assay

NO levels were assayed by measuring the accumulation of stable oxidative metabolites (NOx, nitrite and nitrate) with the sensitive colorimetric Griess reaction, using trichloracetic acid (TCA), Vanadium (III) chloride and Griess reagent, as previously described [36]. For macrophage NO production, peritoneal macrophages were incubated overnight. One hundred microliters of supernatant was treated with 100μL of Griess reagent (1% sulphanilamide, 0.1% naphtylethylenediamine dihydrochloride, and 5% orthophosphoric acid), and the mixture was incubated at room temperature for 5min. The absorbance was measured at 540nm in a microplate reader. The amount of nitrite in the sample was determined using sodium nitrite for the standard curve [37].

#### Arginase activity assays

The tests were carried out using a spectrophotometric assay based on the determination of the production of urea after the addition of L-arginine [38,39]. Bacterial and macrophage lysates were used to measure the effect of selenium on the bacterial and macrophage arginase activities, respectively: the bacterial arginase assay was carried out on a culture without macrophages; the macrophage arginase was performed on removed adherent macrophage cells from the tissue culture plastic after culture in the presence of *S*. *aureus*. Briefly, 25 μL of bacterial or macrophage cell lysate were added to 200 μL aliquot of arginine buffer (10 mM L-arginine, pH 6.4), and the mixture was incubated at 37°C for 60 min. The reaction was stopped by adding 750 μL of acetic acid [40], and the amount of urea generated by arginase [41] was analyzed at 600 nm using a commercial kit (UREA/BUN—COLOR, BioSystems, S.A. Costa Brava 30, Barcelona, Spain). The arginase activity was expressed as nmol urea/mg proteins/1hr.

#### Hydrogen peroxide assay

H_2_O_2_ measurement was carried out spectrophotometrically using commercial kit according to the manufacturer instructions (Sigma-Aldrich, St. Louis, Missouri, USA).

#### Bacterial growth and CFUs count assay

The effect of diverse concentrations of selenium (0, 5, 10, 20, 30 and 40 ng/mL) added to the media on bacterial growth was assayed according to the arginase activity. After culture for one hour with macrophage cells, a volume of 0.2 mL of diluted bacterial suspension was plated on the surface of Chapman medium. Following incubation for 24 hours at 37°C, a counting of bacterial colony forming units (CFUs) was performed using ImageJ software (NIH, USA), as reported [42].

#### Phagocytosis and bacterial killing assays

Tests of phagocytosis and bacterial killing were performed using a bactericidal assay as described in detail elsewhere [43], with some modifications. Assays were made at 0 and 60 minutes on six-well plates containing either a mixture of macrophages and *S*. *aureus* cells with the different concentrations of selenium or bacterial cells alone. The number of viable bacteria was determined microbiologically based on the counting of CFUs on Chapman medium as reported above. The results were expressed as a percentage of phagocytosis and bacterial cells killing, calculated as follows:

$$\text{percentage of phagocytosis}\;=\text{M}0-100\times \frac{\left(\frac{\text{EC}}{\text{bacterial growth}}\right)}{\text{M}0},\;\text{percentage of bacterial killing}=100-100\;\times \frac{\text{M}60}{\text{M}0}$$

with bacterial growth = C60/C0 where C0 and C60 correspond to control sample at 0 minute and 60 minutes respectively. The percentage of viable bacteria = $100\;\times \frac{\text{M}60}{\text{M}0}$. M0 corresponds to mixture assay sample at 0 minute and M60 corresponds to mixture assay sample at 60 minutes. EC, mixture assay sample supernatant after centrifugation.

### Statistical analysis

All data are expressed as the means ± standard error. Non-parametric Mann-Whitney U test was used to compare the differences between two groups. Comparison of more than two independent groups was carried out using Kruskal-Wallis test. Statistical analyses were performed using SPSS 16.0 (Statistical Package for the Social Sciences, SPSS Inc., Chicago, IL, USA) software. A *p*-value less than 0.05 was considered statistically significant.

## Results

### *In vivo* effect of selenium

**Fig 2:**
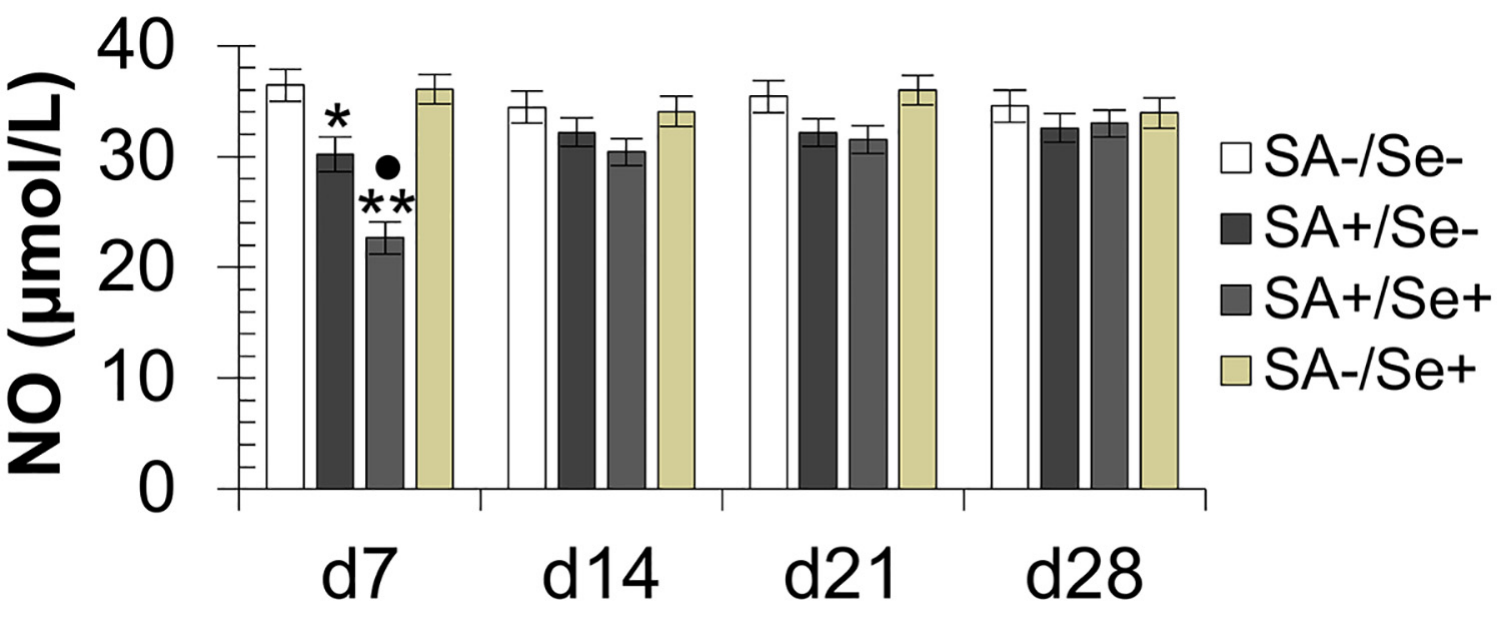
Effect of selenium supplementation on serum nitric oxide measured by colorimetric Griess reaction. SA+/Se+: animals infected by *S*. *aureus* and with oral supplementation by 0.6 mg/kg/day selenium, SA+/Se-: infected animals without selenium supplementation, SA-/Se+: non-infected selenium-supplemented animals, controls: not infected and not supplemented group (SA-/Se-). NO: nitric oxide production [NOx, nitrite (NO_2_-) and nitrate (NO_3_-)]. d7, d14, d21, d28: numbers of days post-infection. The asterisks indicate a significant difference between infected and control animals as follows: •^,^*p < 0.05, **p < 0.01. The black dots indicate the difference between SA+/Se+ and SA+/Se-.

All the clinical and behavioral characteristics of the four animal groups were similar at the beginning of the experiment. Additionally, all the last group of hamsters resumed normal and same habit at the end of the experiment, *i*.*e*. at day 28 post-infection. For both groups of infected animals, we observed that the number of bacteria decreases progressively and significantly from the third day post-infection (*p* < 0.001). Although circulating levels of *S*. *aureus*-specific antibodies were significantly higher in selenium-supplemented animals compared with those not supplemented (S2 Fig, S1 File), the bacterial eradication appeared later in the first week after infection in selenium-supplemented group (*p* < 0.01). At day 7 post-infection, the circulating levels of NO were significantly decreased in infected animals compared to non-infected animals, supplemented or not with selenium (respectively, *p* < 0.01, *p* < 0.05). They remain decreased until the end of the experiment in infected animals compared to non-infected animals, but the difference did not reach statistical significance (*p* > 0.05). Additionally, infected and selenium-supplemented animals have significantly decreased levels of serum NO when compared with infected but not selenium-supplemented animals at day 7 post-infection (*p* < 0.05). In contrast, no significant difference was revealed between the four groups of animals from day 14 post-infection (Fig 2).

### Effect of selenium on peritoneal macrophage activation

**Fig 3:**
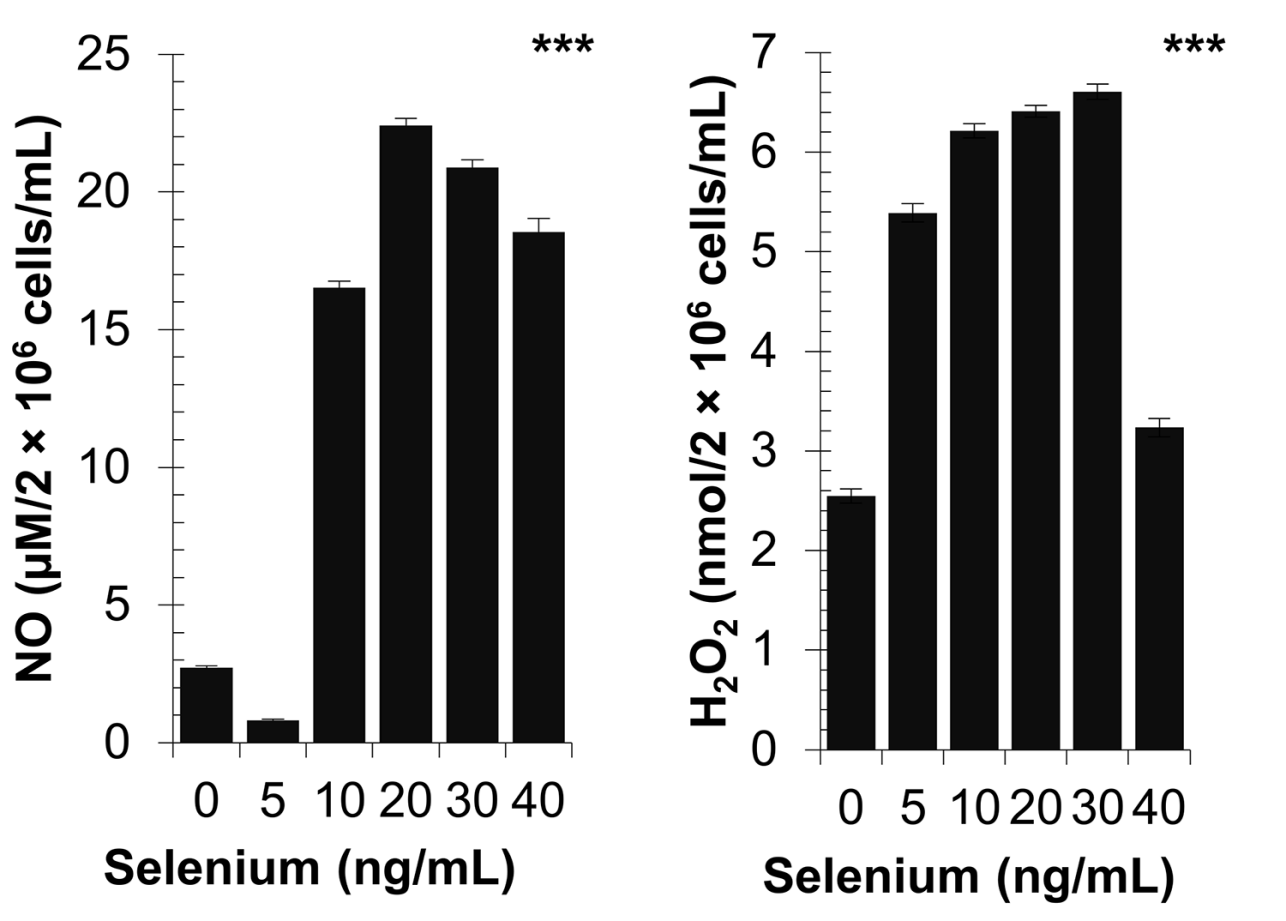
Effects of diverse selenium concentrations on activation of peritoneal macrophage from non-infected and non-selenium-supplemented hamsters cultured in the presence of *S*. *aureus*. The *ex vivo* macrophage activation was performed using the NO production and H_2_O_2_ assays. NO: nitric oxide production [NOx, nitrite (NO2-) and nitrate (NO3-)], H_2_O_2_: hydrogen peroxide. *P*-values are given with significance degree indicated by asterisks. ***p < 0.0001 by Kruskal-Wallis test.

The addition of a low selenium concentration (5 ng/mL) induced a significant decrease in NO production, but significant increase in H_2_O_2_ levels (Fig 3). Additionally, the NO production and H_2_O_2_ levels were significantly increased with increasing concentrations of selenium, but then significantly decreased again at 40 ng/mL; the optimal macrophage activity levels were reached between 20 ng/mL and 30 ng/mL (for both variables, *p* < 0.0001 by Kruskal-Wallis test).

### Effect of selenium on bacterial and peritoneal macrophage arginase activity

**Fig 4:**
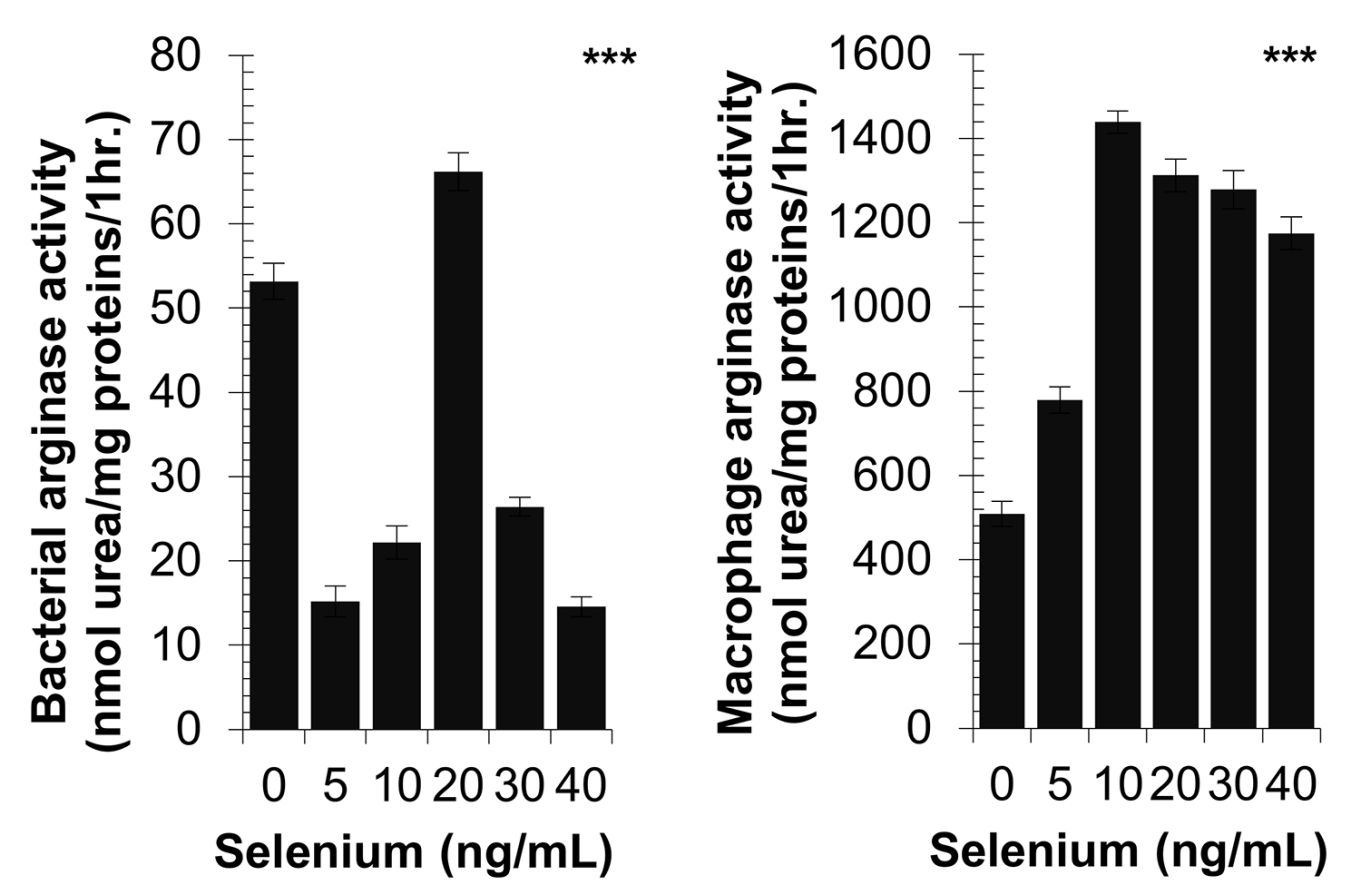
Effect of diverse selenium concentrations on arginase activity of cell lysates from *S*. *aureus* or peritoneal macrophages from non-infected and non-selenium-supplemented hamsters cultured in the presence of *S*. *aureus*. Arginase activity assays were carried out using a spectrophotometric method based on the determination of the production of urea after the addition of L-arginine. *P*-values are given with significance degree indicated by asterisks: ***p < 0.0001 by Kruskal-Wallis test.

The concentration of 5 ng/mL of selenium induced a significant decrease in the activity of the bacterial arginase. This activity varied in the Gaussian form according to selenium concentration and reached a maximum at 20 ng/mL (Fig 4). In contrast, the concentration of 5 ng/mL selenium induced a significant increase in the macrophage arginase activity. This activity also changed as a Gaussian distribution, but reached the optimum at 10 ng/mL. The Kruskal-Wallis test gave *p*-value < 0.0001 for the two comparisons.

### Effect of selenium on peritoneal macrophage NO production/arginase and arginase/NO production ratios

**Fig 5:**
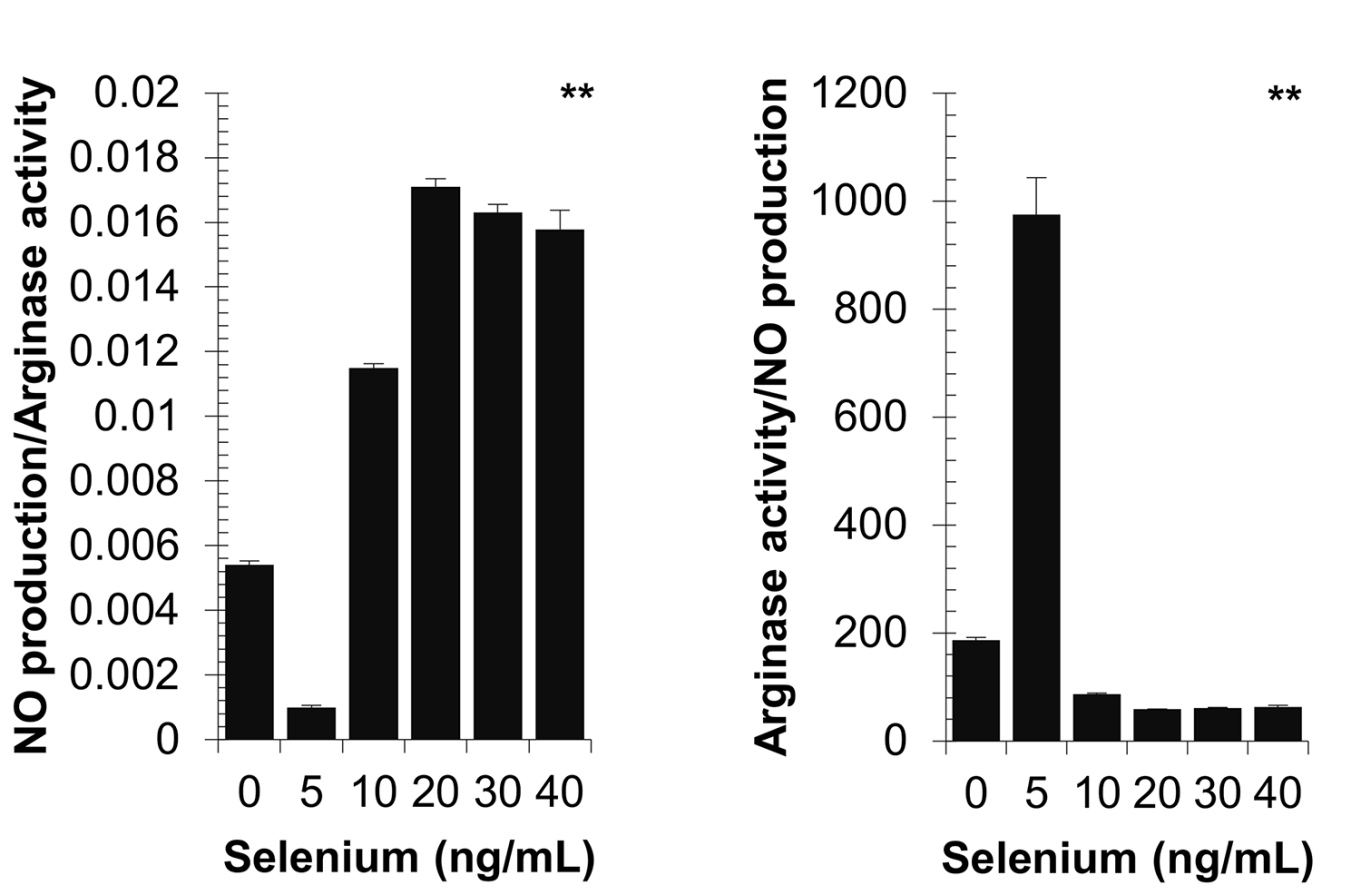
Effect of diverse selenium concentrations on NO production/arginase and arginase/NO production ratios of peritoneal macrophage from non-infected and non-selenium-supplemented hamsters cultured in the presence of *S*. *aureus*. *P*-values are given with significance degree indicated by asterisks. **p < 0.001 by Kruskal-Wallis test.

The NO production/arginase ratio decreased significantly with a low selenium concentration corresponding to 5 ng/mL, and then increased significantly from 10 ng/mL to reach the optimal level at 20 ng/mL (Fig 5). Additionally, NO production/arginase ratio changed as a Gaussian distribution. In contrast, the arginase/NO production ratio reached the optimal level with a concentration of 5 ng/mL selenium, and then decreased significantly from 10 ng/mL to reach a lowest level with a dose of 20 ng/mL selenium. For both comparisons, *p-*value = 0.0001 were obtained by Kruskal-Wallis test.

### Effect of selenium on *S*. *aureus* growth according to the overall arginase activity

**Fig 6:**
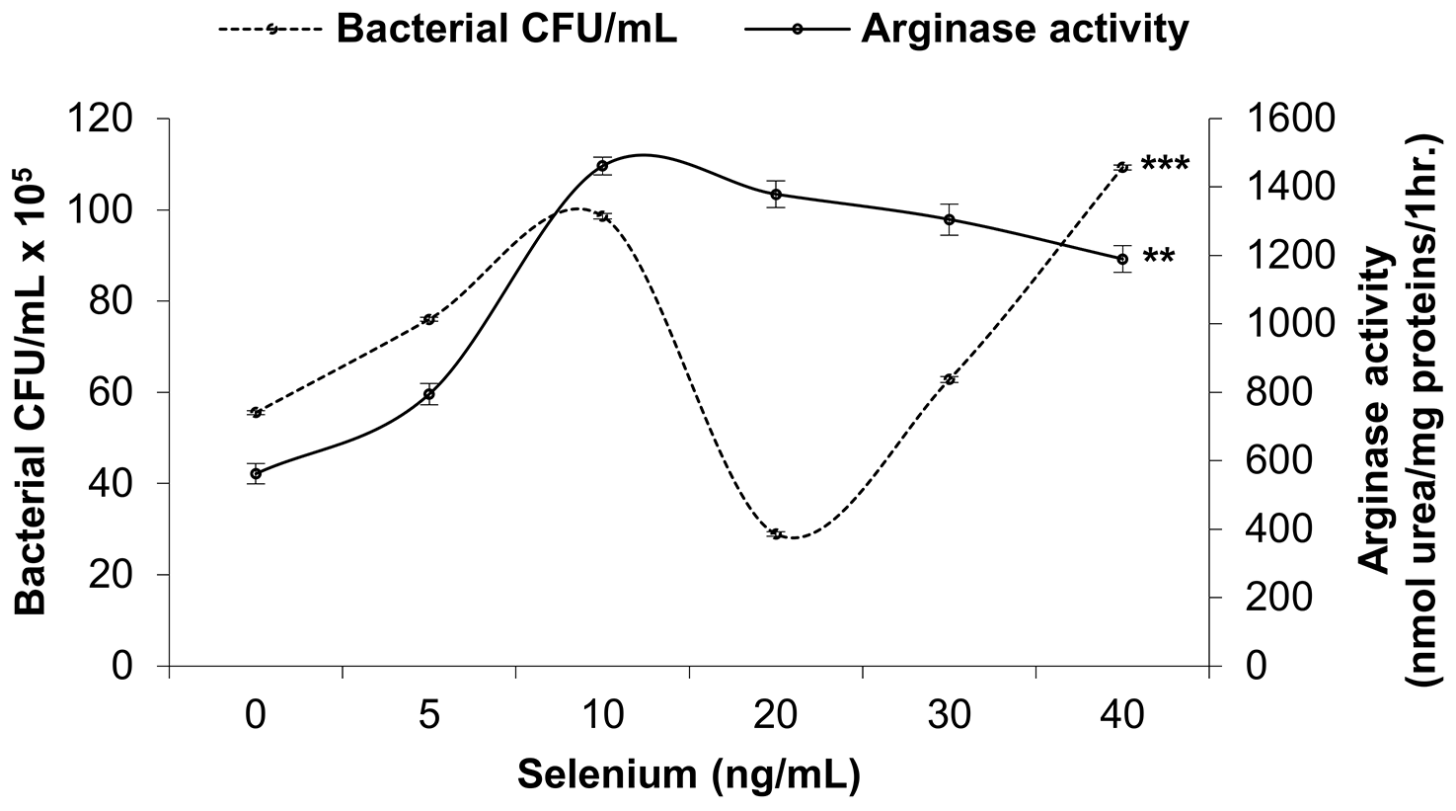
Effect of diverse selenium concentrations on *S*. *aureus* growth according to the overall arginase activity. Bacterial culture was performed on Chapman medium and counting of CFUs was determined using ImageJ software (NIH, USA). Overall arginase activity was determined as the amount of arginase activity of both peritoneal macrophage and bacteria cultured in the presence of diverse concentrations of selenium (0, 5, 10, 20, 30 and 40 ng/mL). CFU: colony forming unit. *P*-values are given with significance degree indicated by asterisks. **p < 0.001, ***p < 0.0001 by Kruskal-Wallis test.

The bacterial growth rate was simultaneously increased with increased of overall arginase activity at selenium concentrations between 0 and 10 ng/mL. From 10 to 20 ng/mL selenium, both the rate of bacterial growth and overall arginase activity decreased; nevertheless, the decrease in the arginase activity does not reach the significance level (respectively, *p* < 0.0001; *p* = 0.120 by Mann-Whitney U test). Between the doses of 20–40 ng/mL selenium, arginase activity significantly decreased (*p* = 0.022), but the *S*. *aureus* growth rate significantly increased (*p* = 0.000). For all comparisons using the Kruskal-Wallis test, *p-*values were < 0.0001 and < 0.001 for bacterial growth and arginase activity, respectively (Fig 6).

### Effect of selenium on peritoneal macrophage phagocytosis and bacterial killing

**Fig 7:**
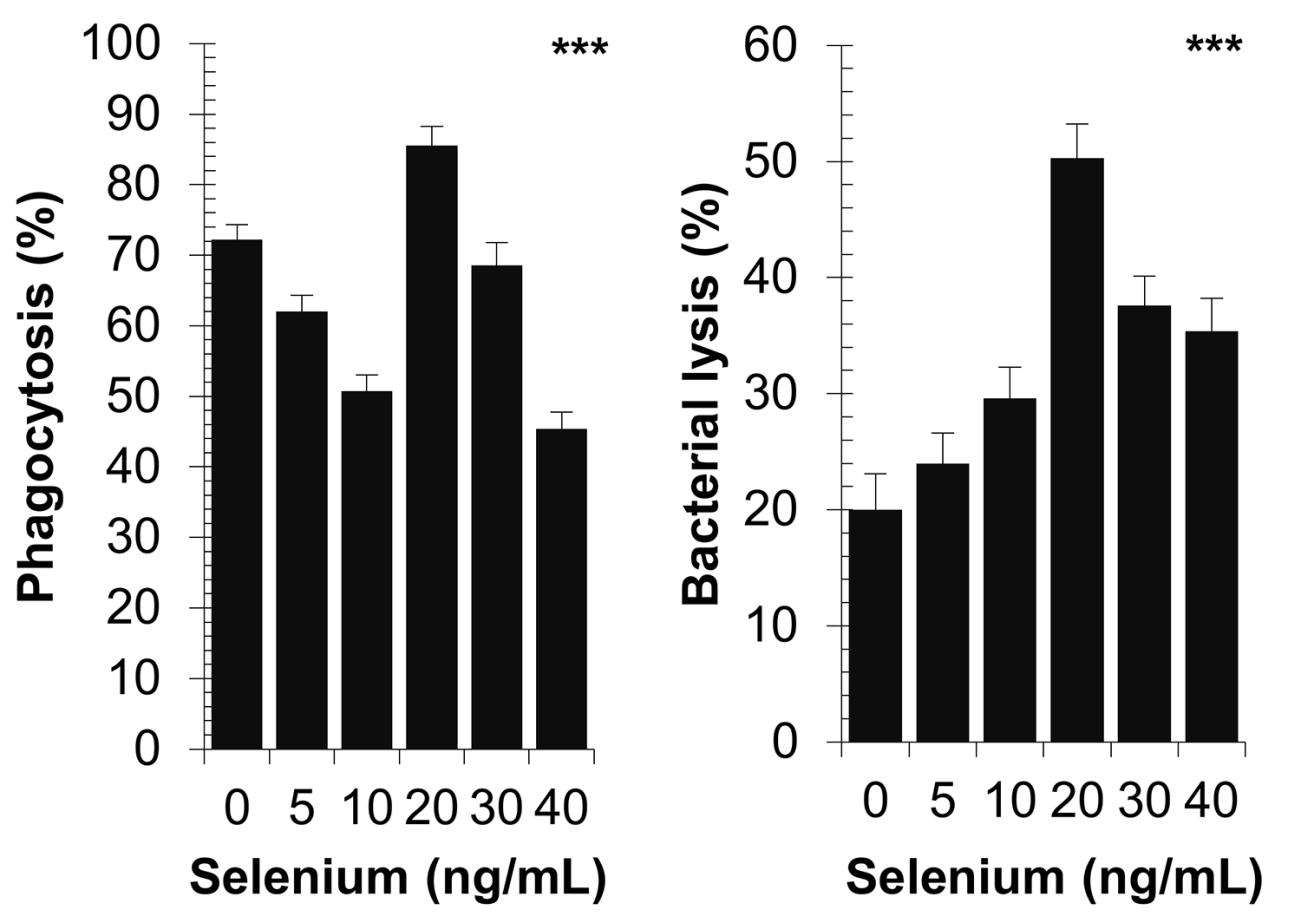
Effect of diverse selenium concentrations on peritoneal macrophage phagocytosis and *S*. *aureus* killing. Macrophages were isolated from non-infected and non-selenium-supplemented hamsters. Assays were made at 0 and 1hr on six-well plates containing either a mixture of macrophages and *S*. *aureus* cells with the differente concentrations of selenium or bacterial cells alone. The results were expressed as a percentage of phagocytosis and bacterial cells killing. *P*-values are given with significance degree indicated by asterisks. ***p < 0.0001 by Kruskal-Wallis test.

Selenium progressively decreased the phagocytic activity in the range of 5 ng/mL to 10 ng/mL selenium; this activity increased significantly to reach an optimal level with a dose of 20 ng/mL selenium (Fig 7). Inversely, selenium gradually increased the activity of bacterial lysis from a dose of 5 ng/mL. Similarly to the phagocytic activity, bacterial lysis reached an optimal level with a dose of 20 ng/mL selenium. The activity of *S*. *aureus* killing by peritoneal macrophage changed as a Gaussian distribution. *P*-value was < 0.0001 for the two comparisons using the Kruskal-Wallis test.

## Discussion

Selenium is the main component of selenoproteins and functions as a redox centre [44]. It is transported in blood with selenoprotein P (SePP) and can be stored as selenomethionine (SeMet) in different tissues and organs, such as liver, kidney, pancreas, heart, brain and spleen. Sodium selenite, a common dietary form of selenium, is an essential micronutrient as well as a toxic trace element in animal and human nutrition.

The strong involvement of selenoproteins in various metabolic pathways may explain the importance of selenium status in several vital functions like immune defense. It has been suggested that it modifies the interactions between macrophages and lymphocytes or acts as an antioxidant on the cells involved in immunological reactions [45]. It also acts as a modulator of inflammatory and immune responses by helping to control peroxide concentration at sites of inflammation, and by decreasing leukotriene synthesis and stimulating cellular immunity [46]. Additionally, selenium: i) plays an important role in the regulation of the expression of pro-inflammatory genes and cytokines and of their receptors on immune cells [47], ii) can suppress the expression of cyclooxygenase (COX)-2, prostaglandin E2 (PGE2), interferon (IFN)-regulatory factor 3 (IRF3) and tumour necrosis factor (TNF)-α, and modulate both myeloid differentiating factor 88 (MyD88)- and TIR-domain-containing adapter-inducing interferon-β (TRIF)-dependent signaling pathways of toll-like receptors (TLRs) leading to decreased inflammatory gene expression [48], iii) can reduce markers of inflammation in patients with systemic inflammatory response syndrome (SIRS) and sepsis [49], and iv) can mediates inhibition of the activation of the transcription factor NF-kB, which regulates genes that encode inflammatory cytokines [50]. In this context, our study aimed at investigating the role of selenium supplementation on macrophage activity during *S*. *aureus* infection and significant effects of selenium were demonstrated on macrophage activation, phagocytosis and bacterial killing.

### *In vivo* effect of selenium on serum nitric oxide

The activation of the immune system by bacterial products induces the release of reactive oxygen or nitrogen species. Among these, NO is useful in the initial phase of the innate immune response by acting as a proinflammatory molecule and can act as a potent microbicidal agent, which prevents the development of a number of microorganisms, including viruses, bacteria, fungi, and parasites. The antimicrobial properties of NO can be the result of several actions on DNA, proteins and lipids. Besides these antimicrobial properties, it can also induce apoptosis and cell cycle arrest [51].

On the other hand, NO is also critically involved in the regulation of a number of diverse biological processes, including innate immunity and immunological host defense against invading pathogens and wound healing [52]. It is produced from L-arginine by the enzyme NOS from different cells. Various inflammatory cytokines or TLR agonists may induce the expression of iNOS. The production of NO particularly through expression of the isoform iNOS induced by inflammatory cytokines or TLR agonists occurs in a variety of microbial infections as an effective antimicrobial agent. Several studies have shown that serum NO levels were significantly higher in many animal species during inflammation due to microbial infection, and that these levels decrease under anti-inflammatory treatment [53,54]. Consequently, NO can be used as a clinical marker to evaluate the efficacy of anti-inflammatory drugs. Contrarily to these previous observations, the serum NO levels were significantly decreased in early *S*. *aureus* infection in our study. Moreover, selenium supplementation induces further decreasing of NO levels, which may reflect the role of selenium as a modulator of inflammatory responses. Thereby, our results corroborate those of previous researches showing the inhibitory effect of selenium on the levels of NO production [48,55].

The inhibition of bacterial growth was shown less important during the first week of infection in selenium-supplemented animals compared with those not supplemented. Although *S*. *aureus*-specific antibodies levels were higher in supplemented animals compared with those of not supplemented, those of NO were decreased. Altogether, these results suggested that the process of *in vivo* eradication of *S*. *aureus* would be much more effective in the presence of mediators of non-adaptive immunity, like NO.

### Effect of selenium on peritoneal macrophage activation

Macrophages are considered as key components of the innate immune system, but also as orchestrator of adaptive immunity [56]. They can directly kill microorganisms and promote their clearance by phagocytosis. Their activation and microbicidal function are characterized by the release of many effectors, like NO and H_2_O_2_ [57,58].

H_2_O_2_ generated from activated macrophage is produced by respiratory/oxidative burst based on the increased activation of membrane-associated NADPH oxidase complexes [59], and may be induced by pathogens opsonized with complement factor C3b/iC3b, IgG-coated bacteria to Fc receptors, C3a- and C5a-mediated chemotaxis, etc. [60,61]. It can enhance the bacterial killing power, but can also have adverse effects on the cells and the surrounding healthy tissue. H_2_O_2_ also functions as a signaling agent, particularly in higher organisms [62]. It is freely diffusible within and between cell membranes, but can be decomposed biologically by catalase, produced by some bacteria, such as *S*. *aureus*, to protect against the host respiratory burst [63], and consequently to evade hosts' immune defense to a certain extent.

It has been recently demonstrated that optimal selenium concentration is critical for macrophage activation and resolution of inflammation [64]. We observed that selenium affects macrophage activation in a dose-dependent manner, a low dose impairing NO production but increasing H_2_O_2_ levels. These results are in accordance with previous studies showing that during infection, antioxidant nutrients commonly included in the diet such as selenium are absolutely necessary to regulate the reactions that release free radicals [65]. Indeed, selenium affects NF-κB activation, which regulates genes that encode inflammatory cytokines [66], may be able to downregulate the LPS-induced expression of iNOS and other proinflammatory genes in macrophages, and its deficiency increases the expression of iNOS [67,68].

### Effect of selenium on bacterial arginase activity

Among many virulence factors, arginases secreted by *S*. *aureus* can compete with iNOS for their common substrate, the amino acid L-arginine (L-Arg) [69]. Consequently, the NO production in activated macrophages will be reduced, since its translation is strongly dependent on the availability of L-Arg in the macrophage. The decreased levels of NO then influence negatively on bactericidal activity and phagocytosis, and impact the expression of an NO-inducible L-lactate dehydrogenase (Sa-LDH-1) by *S*. *aureus* [70]. This ultimately results in improved resistance of *S*. *aureus* to oxidative stress, increased growth, bacterial survival and persistence.

We showed a dose-dependent effect of selenium on the activity of both bacterial and macrophage arginase. A low concentration of selenium of 5 ng/mL induced a strong alteration of the bacterial arginase activity, thereby counteracting the effects described above for this virulence factor.

### Effect of selenium on peritoneal macrophage arginase activity and NO production/arginase balance

The lower concentration of selenium tested (5 ng/mL) also induced a significant decrease in the concentration of NO production, a significant increase in the macrophage arginase activity, and, consequently, a significant decrease in NO production/macrophage arginase activity ratio. In contrast, high concentrations of selenium could allow optimum expression of NO and a sharp decrease in the arginase activity. It is therefore important to remember that arginases and various NOS compete for the same substrate L-Arg and that their enzymatic activities can be inhibited reciprocally [71–73]. The use the NO production/arginase balance and not the results of NO production or arginase activity alone to evaluate the bactericidal efficacy against the bacterial escape is therefore essential.

Nevertheless, the concentration of NO produced by macrophages *in vitro* may not fully reflect the concentration of NO *in vivo*. The differences between the *in vivo* and *in vitro* assays could partly be due to dose effects, selenium metabolism and pharmacology.

### Effect of selenium on *S*. *aureus* growth according to the overall arginase activity

Several new therapeutic approaches using various selenium compounds and nanoparticles have been developed to inhibit *S*. *aureus* growth and pathogenesis. Among them, selenium nanoparticles synthesized from sodium selenite were shown to inhibit *S*. *aureus* growth *in vitro* [74,75]. Selenium concentration that may be delivered by such nanoparticules at the infection site has to be considered with caution because we showed that dose-dependent effects of selenium on *S*. *aureus* growth, 20 ng/mL selenium causing both significant growth inhibition of *S*. *aureus* and decrease of arginase activity while doses higher than 20 ng/mL could, on the contrary, increase bacterial colony number despite their ability to decrease the overall arginase activity.

### Effect of selenium on peritoneal macrophage phagocytosis and bacterial killing

We showed that selenium supplementation can enhance phagocytosis and bactericidal capacity in a dose-dependent manner reaching a maximum level for a 20 ng/mL selenium dose. However, mechanisms impacted by selenium could not be totally deciphered because of the complexity and diversity of involved factors. Indeed, several well-defined surface components present in most clinical isolates of *S*. *aureus* also prevent opsonization and phagocytosis, such as polysaccharide capsule [76]. Additionally, most strains produce chemotaxis inhibitory protein [77] that can delay leukocyte migration toward the site of infection [78] and secrete various C3 complement inhibitors [79]. Finally, the ability of *S*. *aureus* to form three-dimensional biofilms during the infectious cycle [80] can also prevents phagocytosis [81]. Currently, the impact of selenium on these different factors remain largely unknown despite selenium has been shown to influence several cells or functions involved in innate immunity like lymphokine activated killer cells and macrophages, the tumor cytotoxicity by mouse macrophages, and the phagocytic function of neutrophils and macrophages [82–85].

## Conclusions and Future Prospects

Selenium supplementation can enhance the *in vivo* control of immune response to *S*. *aureus*. To the best of our knowledge, we reported here for the first time that during the initial phase of infection, the *in vivo* bacterial eradication processes would strongly be based on the mediators of the non-adaptive immunity, such as NO. In fact, during this phase, the selenium administration induced a decrease in circulating levels of NO, and simultaneously a low inhibition of bacterial growth. In the *ex vivo* experiments, we observed that selenium acts in a dose-dependent manner on macrophage activation, phagocytosis and bacterial killing, suggesting that inadequate doses may cause a loss of macrophage bactericidal activities. These results warrant further investigations and among others, it would be of particular interest to: i) complete arginase activity study by qPCR-based expression or western immunoblotting assays, ii) conduct a kinetic study in hamsters using different doses of sodium selenite, iii) check whether it would be possible to replicate our results using other cellular models of macrophage from different origins, such as bone marrow and spleen, and iv) monitor bacterial load in peritoneal fluid samples and follow disease progression and outcome. *In fine*, clinical evaluation will still be required to determine the *in vivo* impact and optimal conditions of selenium administration in patients with *S*. *aureus* infection.

## Supporting Information

S1 Fig *mecA* gene amplification for the *Staphylococcus aureus* isolate. Lanes 1 to 4, positive control (amplification product of 528 pb), GeneRuler 100-bp DNA Ladder (Fermentas), negative control, methicillin-suceptible *S*. *aureus* (MSSA) isolate. Positive and negative controls are clinical isolates of *S*. *aureus*, respectively *mecA*+ and *mecA*- (Laboratoire de Bactériologie, Centre Hospitalier Régional Universitaire de Montpellier).
(TIF)

S2 Fig Effect of selenium supplementation on circulating levels of immunoglobulins and specific antibodies. SA+/Se+: infected and selenium-supplemented animals, SA+/Se-: infected animals without selenium supplementation, Controls: not infected and not supplemented group (SA-/Se-), Sp: specific immunoglobulin M (SpIgM) or G (SpIgG). *p < 0.05.
(TIF)

S1 File Supplementary Results. (DOCX)
