# Supplementary figures and images for: Macrophage Bactericidal Activities against Staphylococcus aureus Are Enhanced In Vivo by Selenium Supplementation in a Dose-Dependent Manner
Source: PLoS One. 2015 Sep 4;10(9):e0135515. doi: 10.1371/journal.pone.0135515 (PMC4560415; doi:10.1371/journal.pone.0135515)

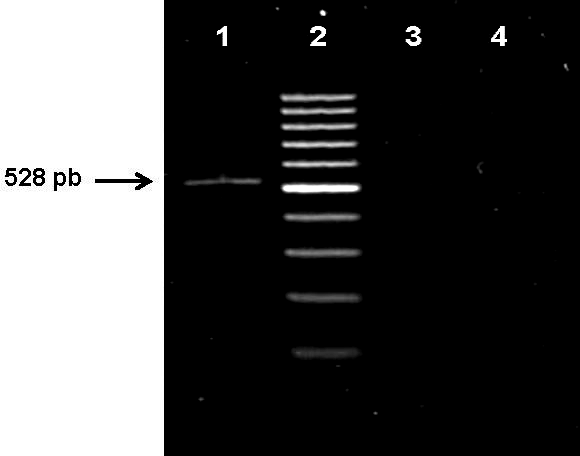

Supplement: S1 Fig — Lanes 1 to 4, positive control (amplification product of 528 pb), GeneRuler 100-bp DNA Ladder (Fermentas), negative control, methicillin-suceptible S. aureus (MSSA) isolate. Positive and negative controls are clinical isolates of S. aureus, respectively mecA+ and mecA- (Laboratoire de Bactériologie, Centre Hospitalier Régional Universitaire de Montpellier). (TIF) [file pone.0135515.s001.tif]

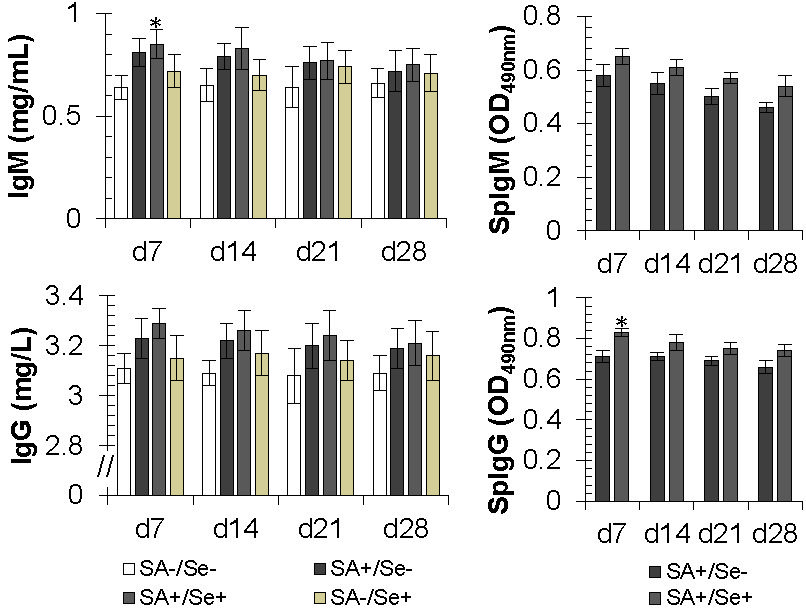

Supplement: S2 Fig — SA+/Se+: infected and selenium-supplemented animals, SA+/Se-: infected animals without selenium supplementation, Controls: not infected and not supplemented group (SA-/Se-), Sp: specific immunoglobulin M (SpIgM) or G (SpIgG). *p < 0.05. (TIF) [file pone.0135515.s002.tif]
