# Supplementary material for: Macrophage Bactericidal Activities against Staphylococcus aureus Are Enhanced In Vivo by Selenium Supplementation in a Dose-Dependent Manner
Source: PLoS One. 2015 Sep 4;10(9):e0135515. doi: 10.1371/journal.pone.0135515 (PMC4560415; doi:10.1371/journal.pone.0135515)
Supplement: S1 File — (DOCX) [file pone.0135515.s003.docx]

**Supplementary results**

**Effect of selenium on serum immunoglobulins and specific antibodies**

Supplementary Figure 2 shows that serum levels of IgM and IgG were higher in infected animals from seven until twenty eight days post-infection compared to non-infected animals, independently of selenium supplementation. Nevertheless, the difference between them was not significant (for all comparisons, *p* > 0.05). Additionally, circulating levels of *S. aureus*-specific IgM (SpIgM) and IgG (SpIgG) antibodies were increased from 7 until 28 days after infection in selenium-supplemented animals as compared with non-selenium-supplemented animals; whereas, the significant level was reached only for SpIgG at day 7 after infection (*p* = 0.010).

**Discussion**

In addition to its role in the inflammatory response, nutritional supplementation of selenium can also play a role in boosting immunity, especially in the regulation of cytokines expression and enhancing Th1 immunity, which is required for the differentiation of activated T cells into cytotoxic T cells [1], but also in the humoral response by stimulating the production of antibodies by auxiliary activated B cells [2,3].

The immunoglobulin production based on selenium intakes have been evaluated by different authors. Therefore, the intramuscular injection of vitamin E and selenium, or the single oral administration of selenium, increases the production of immunoglobulins in calves after injection of egg lysozyme [4]. These data are fully consistent with ours. However, there are heterogeneous results in terms of the production of immunoglobulins, depending on the animal species, the specificity of the antibody and the nature of the infectious agent. Therefore, similarly to our results about specific antibodies aginst *S. aureus*, it has been observed in sheep and lambs that the selenium supplementation in feed led to an increased antibody production against *Clostridium tetani* [5] or PI3 virus and *Corynebacterium ovis* [6]. In contrast, in vitamin E and selenium doubly deficient mice infected with *Citrobacter rodentium*, the antigen-specific IgG or IgM production is not affected by vitamin E and selenium administration [7].

We observed that at day 7 post-infection, the IgM and IgG titers reach their maximum. So, during the normal course of infection, the pathogen proliferates and reaches the threshold level of induction of the adaptive immune response. Over time, most of the effector T cells die, and antibody levels gradually decline, because the antigens that induced the immune response are no longer present at the level needed to sustain it. This mechanism would be considered as a feedback loop regulating immune response [8].

**References**

1. Hoffmann FW, Hashimoto AC, Shafer LA, Dow S, Berry MJ, et al. Dietary selenium modulates activation and differentiation of CD4+ T cells in mice through a mechanism involving cellular free thiols. J Nutr 2010; 140: 1155-1161. doi: 10.3945/jn.109.120725.

2. Maggini S, Wintergerst ES, Beveridge S, Hornig DH. Selected vitamins and trace elements support immune function by strengthening epithelial barriers and cellular and humoral immune responses. Br J Nutr 2007; 98 Suppl 1: S29-35.

3. El-Shenawy NS, Al-Harbi MS, Hamza RZ. Effect of vitamin E and selenium separately and in combination on biochemical, immunological and histological changes induced by sodium azide in male mice. Exp Toxicol Pathol 2015; 67: 65-76. doi: 10.1016/j.etp.2014.10.005.

4. Swecker WSJr, Eversole DE, Thatcher CD, Blodgett DJ, SchurigGG, et al. Influence of supplemental selenium on humoral immune responses in weaned beef calves. Am J Vet Res 1989; 50: 1760-1763.

5. Moksnes K, Larsen HJ, Øvernes G. Immune responses as parameters for selenium tolerance determination in sheep. In: Hurley LS, Keen CL, Lénnerdal B, Rucker RB, editors. Trace Elements in Man and Animals – TEMA 6, Vol 6. New York and London: Plenum Press; 1988. pp. 91-93.

6. Larsen HJ, Moksnes K, Overnes G. Influence of selenium on antibody production in sheep. Res Vet Sci 1988; 45: 4-10.

7. Smith AD, Botero S, Shea-Donohue T, Urban JF Jr. The pathogenicity of an enteric Citrobacter rodentium infection is enhanced by deficiencies in the antioxidants selenium and vitamin E. Infect Immun 2011; 79: 1471-1478. doi: 10.1128/IAI.01017-10.

8. Janeway CA, Murphy K, Travers P, Walport M. Immunobiologie. 3rd ed. Bruxelle: Groupe De Boecks.a.; 2009.
